# Directed motivational currents as a framework for mutual-benefit peer teaching in foreign language education

**DOI:** 10.3389/fpsyg.2025.1714697

**Published:** 2025-12-04

**Authors:** Shengnan Ma

**Affiliations:** Department of Foreign Languages and Literatures, Tsinghua University, Beijing, China

**Keywords:** mutual-benefit teaching model, directed motivational currents, foreign language teaching, non-profit platform, education

## Abstract

This study applies the Directed Motivational Currents (DMCs) framework to a mutual-benefit and student-led foreign language teaching model at a Chinese university. Eight language majors volunteered as peer instructors and 60 undergraduates enrolled as beginners of French, Japanese, Korean, or German. Drawing on the four DMCs dimensions (vision-based goals, triggering factors, facilitative structure, positive emotionality), the program offered weekly classes supported by quizzes, homework, and participation evaluations. Data from pre- and post-course questionnaires, classroom assessments, and semi-structured interviews were analyzed using a mixed-methods approach. Quantitative results showed that most learners were driven by intrinsic interest, maintained participation, and improved their quiz performance over the semester. Qualitative themes highlighted vivid learner and teacher visions, diverse triggers, supportive routines, and positive emotions for both groups. Overall, the findings suggest that a DMCs-informed peer-teaching model is a feasible non-profit approach for sustaining motivation and expanding equitable access to foreign language learning in higher education.

## 1 Introduction

In an increasingly globalized world, frequent communication, cultural exchange, and economic cooperation make multilingual ability a critical skill for career advancement, academic opportunities, international travel, intercultural understanding, and personal enrichment as well. As demand for multilingual talent rises, more university students choose to learn additional foreign languages, driven by diverse personal and professional goals. In Chinese universities, English majors are typically required to study a second foreign language, but non-English majors often lack comparable opportunities, even when they are highly interested in learning an extra language for work, study, or leisure. To address this inequity, the present study proposes a peer-teaching initiative in which high-achieving senior students from language departments volunteer as instructors to provide a cost-free foreign language learning platform. In this model, one student-teacher supports several peers in their target language learning through stable teaching-learning partnerships.

Recent work on English language teaching in Hong Kong higher education has shown how the rapid shift to technology-mediated and online delivery has reshaped teaching culture and teacher-learner mediation, thereby increasing the need for clearly structured and student-centered designs (Lo and Chan, 2022). Building on this insight, the present peer-teaching initiative seeks to provide a structured and student-led environment where interaction and support are organized around stable partnerships rather than formal course enrolment. However, such initiative faces challenges. Insufficient motivation on the part of either teachers or learners can undermine participation and threaten the sustainability of the platform; the absence of a solid theoretical framework also weakens its instructional effectiveness. To solve these problems, this study adopts the construct of Directed Motivational Currents (DMCs), i.e., intense and sustained motivational surges that support long-term goal pursuit (Dörnyei et al., 2016). Motivation is a key determinant of second language learning success (Dörnyei and Ryan, 2015), and the DMCs framework offers both conceptual grounding and practical strategies for maintaining engagement over time.

The significance of this study lies in applying the DMCs framework to a teaching model that is simultaneously pedagogically sound and socially influencing. By drawing on foreign language majors’ expertise and offering them meaningful teaching opportunities, the model promotes reflection, skill development, and professional growth. Meanwhile, it provides non-English majors with access to quality instruction that would otherwise be unavailable. If proven effective, the model can be extended to other disciplines and institutional contexts as a replicable framework for student-led, motivation-informed educational initiatives.

## 2 Literature review

Internal contributing factors to second language (L2) acquisition include motivation (Ellis, 2015), which can explain individuals’ choices to undertake specific actions, their persistence in performing them, and the effort they expend to achieve them (Dörnyei and Ushioda, 2013). Motivation for language learning has inspired a growing body of research (e.g., Al-Hoorie and Mac Intyre, 2019; Ghazvini and Khajehpour, 2011; Klimova, 2011). It plays a significant role in second language learning success and achievement (Dörnyei and Ryan, 2015), functioning as an individualized trait that enables learners to persist long enough to master the L2 regardless of their language aptitude or cognitive characteristics (Dörnyei, 2001; Masgoret and Gardner, 2003).

The concept of Directed Motivational Currents (DMCs), proposed by Dörnyei et al. (2014), is defined as “an intense motivational drive, or surge, which is capable of stimulating and supporting long-term behavior such as the learning of an L2” (Dörnyei et al., 2016: 18). It has considerable application value in foreign language teaching and research and can enhance both the instructional quality of foreign language classes and students’ learning efficiency. DMCs are supported by a growing body of research (e.g., Henry et al., 2015; Safdari and Maftoon, 2017; Zarrinabadi and Tavakoli, 2017). The DMCs framework involves four dimensions, i.e., goal/vision-orientedness, a triggering factor and launch, a facilitative structure, and positive emotionality (Dörnyei et al., 2016).

Firstly, goal/vision-orientedness directs an individual’s motivational current (Peng and Phakiti, 2022), which “transports the individual towards a highly valued end” (Dörnyei et al., 2016). Dörnyei and Kubanyiova (2014) define a vision as a goal enriched by the imagined experience of its realization. Secondly, the launch of a triggering factor refers to a necessary stimulus for task engagement, including ownership of the task and confidence in completing it. When such stimuli occur, latent motivational energy may be released (Henry et al., 2015). Thirdly, a facilitative structure supports behavioral routines and progress checks (Dörnyei et al., 2016). These routines help learners develop semi-automatic behaviors in language use, while progress checks provide ongoing performance evaluations (Peng and Phakiti, 2022; Zarrinabadi et al., 2019). Fourthly, the potential of achieving the envisioned goal and the accomplishment of sub-goals can result in positive emotionality (Dörnyei et al., 2016). Although some tasks along a DMCs pathway may not be inherently enjoyable (Muir and Gümüş, 2020), such as sacrificing time for language classes (Safdari and Maftoon, 2017), they can still support long-term motivation. Proximal sub-goals also serve as markers of personal progress and stimulate positive feedback (Bandura and Schunk, 1981; Sak, 2019).

Related research in higher education has also emphasized the role of emotional regulation, self-efficacy, and perceived control in sustaining engagement in English courses designed with flipped or hybrid elements. Drawing on focus-group feedback from Hong Kong university students, Lo (2023) illustrated that carefully structured hybrid designs, combining online preparation with interactive in-class activities, could function as an “emotional bridge” supporting positive affect, autonomy, and confidence, which in turn predicted productive engagement and achievement. These findings complement the DMCs emphasis on facilitative structure and positive emotionality through clarifying how course design can scaffold learners’ emotions and sense of agency in university language classes.

Jahedizadeh and Al-Hoorie (2021) conducted the systematic review of DMCs studies and found that most empirical investigations were small-scale and context-specific. Their review highlights the need for pedagogically grounded and context-sensitive applications of the framework, especially in settings where learner well-being must be protected. Building on this line of work, Bui (2023) explored DMCs in the context of group projects among Vietnamese university students, showing how collaborative structures and shared project goals contribute to group-level motivational currents sustaining effort over time. These findings suggest that the careful design of structured and collaborative learning environments can promote DMCs-informed motivational patterns in higher education.

DMCs exist alongside influential motivational theories such as Self-Determination Theory (SDT; Deci and Ryan, 1985; Ryan and Deci, 2000), the L2 Motivational Self System (L2MSS; Dörnyei, 2009), and Complex Dynamic Systems Theory (CDST; Larsen-Freeman and Cameron, 2008; Hiver et al., 2022). SDT emphasizes autonomy, competence, and relatedness in fostering intrinsic motivation, whereas L2MSS focuses on future self-images, especially the Ideal L2 Self. CDST conceptualizes motivation as a dynamic, emergent system arising from multiple interacting variables over time. The DMCs perspective contributes uniquely by highlighting phases of goal-directed motivation and the formation of intense motivational surges. In this study, DMCs are adopted as the primary theoretical framework because they inspire both instructional design and the structuring of learner experiences in a student-led program, offering concrete guidance for practice.

University students exhibit diverse motivations for learning languages, ranging from personal interest to institutional requirements. Klimova (2011) found that learners may be driven by curiosity, academic obligations, or family expectations, factors that function as motivational triggers even in passive learning contexts. With regard to mutual benefit, partnership is a fundamental strategy through which participants meet their needs by sharing resources, capabilities, and skills (Pillay et al., 2014). Partnership is defined as a mutually beneficial relationship entered into by two or more parties in order to achieve shared goals (Owen and Grealish, 2006). Identifying mutual benefits is the foundation for establishing sustainable partnerships and may also be used as a criterion for evaluating partnership programs (Didion et al., 2013; Nabavi et al., 2012).

In addition to the motivational literature, the present study is informed by foundational research on peer learning and peer tutoring in higher education. Peer-assisted learning has a long tradition in this field, with Topping (2005) showing that structured peer tutoring arrangements can yield cognitive, metacognitive, and social gains for both tutors and tutees. Falchikov (2003) further emphasizes that learning together in peer-based contexts promotes shared responsibility, active engagement, and deeper conceptual understanding. Similarly, Boud et al. (2014) argue that peer learning in higher education is most effective when it is designed as a reciprocal process in which students learn with and from each other, thus enhancing autonomy, reciprocity, and reflective practice. On the basis of these perspectives, the present program can be understood as a peer tutoring model that is specifically organized around mutual benefit, i.e., student-teachers develop their linguistic and pedagogical competencies, while student-learners gain access to language instruction that would otherwise be out of reach. By integrating these peer learning principles with the DMCs framework, the study positions its mutual-benefit teaching model within a well-established pedagogical tradition while adding a motivationally focused lens.

From a content-based instruction perspective, a recent study in Hong Kong has also shown that authentic and literature-based tasks can raise motivation and perceived learning gains among undergraduates. In a mixed-methods study of a general-education course on contemporary fiction, Lo and Shi (2024) showed that reading full-length novels and writing extended responses in English led students to perceive improvements in critical thinking, analytical writing, and overall English proficiency, together with high levels of engagement and enjoyment. The results indicate that content-rich and meaning-focused tasks can make language learning more academically rewarding, supporting the present study’s emphasis on meaningful tasks, formative feedback, and students’ active engagement within a peer-teaching model.

Nevertheless, no previous research has applied the DMCs framework to the design and evaluation of a reciprocal, non-profit peer-teaching model that enables students to learn additional foreign languages through partnerships. The present study addresses this gap by developing such a model, which provides non-English majors with access to foreign language learning opportunities while offering student-teachers meaningful opportunities to develop instructional skills. By grounding the program in the DMCs framework and situating it within broader motivational and pedagogical theory, the study seeks to contribute to the motivational literature with a student-led, socially meaningful example of DMCs-informed pedagogy.

## 3 Method

### 3.1 Participants

This study involved a total of 68 participants recruited from a university in China, including eight student-teachers and sixty learners. Recruitment notices were distributed through campus announcements and online bulletin boards. The student-teachers were female undergraduate students aged between 20 and 22, majoring in French, Japanese, Korean, or German, with two representatives from each language. These individuals were selected based on their strong academic performance. They were recommended by faculty members for their linguistic proficiency and teaching potential, and then self-enrolled in the project after being informed of its aims and procedures.

The learner group consisted of 60 non-language-major undergraduates (35 males and 25 females) aged 18 to 22, with 15 students assigned to each language group. All learners met the inclusion criterion of not having prior formal education or self-directed learning experience in the respective target language. Learners filled in an online registration form indicating their preferred target language(s). In accordance with the ethical principles outlined in the Declaration of Helsinki, all participants provided informed consent before participating in the study. The anonymity and confidentiality of the participants were guaranteed, and participation was completely voluntary. To provide an overview of the learner sample, Table 1 summarizes the main demographic and motivational characteristics of the 60 learners, including gender, target language, primary learning goals, attendance patterns, and self-reported learning difficulties.

**Table 1:** Learner demographics, motivational orientations, and attendance (*N* = 60).

Variable	Category	n	%
Gender	Male	35	58.3
	Female	25	41.7
Target language	French	15	25.0
	Japanese	15	25.0
	Korean	15	25.0
	German	15	25.0
Primary learning goal	Curiosity/interest	37	61.7
	Career-related goals	13	21.7
	Social recognition/praise	10	16.7
Attendance frequency	Never absent	18	30.0
	Occasionally absent	30	50.0
	Frequently absent	12	20.0
Main reported learning difficulty	Lack of effort	33	55.0
	External constraints (time, workload)	19	31.7
	No major difficulties	8	13.3

For the qualitative phase, a subset of 20 learners was invited for pre- and post-course interviews, sampled purposively to represent different target languages, genders, attendance patterns, and primary motivational orientations.

### 3.2 Materials

Instructional materials were designed for complete beginners and developed collaboratively by the student-teachers and university faculty. Before the program began, student-teachers completed a two-week training course on pedagogical methods, curriculum design, and classroom management. All courses were conducted in-person in equipped classrooms using audiovisual teaching aids. Each course spanned one academic semester (four months), consisting of weekly sessions comprising two 50-min class periods separated by a 20-min break. To evaluate student goals, motivation, and post-course reflections, pre-course and post-course questionnaires and semi-structured interview protocols were designed. Additionally, formative assessment tasks (quizzes, etc.) were also incorporated, allowing a clearer description of how learning outcomes were measured.

#### 3.2.1 Questionnaires

A pre-course questionnaire was administered at the beginning of the semester and a post-course questionnaire administered at the end. The pre-course questionnaire consisted of 10 items organized into three sections. These covered demographic information (e.g., major, year of study, previous language learning experience), learning goals and motivational orientations, and anticipated challenges and resources. The post-course questionnaire contained 10 items focusing on course experience and attendance, perceived outcomes and difficulties, and emotional responses. All questionnaire items, including single-choice, Likert-scale, and open-ended items, are reproduced in full in Appendix A (pre-course) and Appendix B (post-course). The pre- and post-course questionnaires were used descriptively at the item level, and no composite scales were constructed. Two professors in applied linguistics reviewed and revised the items for clarity.

#### 3.2.2 Interview protocols

Semi-structured interview guides were developed separately for learners and student-teachers. Learner interviews (pre- and post-course interviews) focused on their reasons for joining the program, key experiences that influenced their motivation (for example, weekly quizzes and homework), emotional responses during the course, and overall views on the peer-teaching format. Student-teacher interviews explored their motivations for volunteering, how preparing and delivering lessons affected their own learning, strategies for maintaining learner engagement, and suggestions for future improvements. All interviews were conducted in Mandarin, audio-recorded with consent, and transcribed verbatim for qualitative analysis. The complete learner and student-teacher interview protocols are provided in Appendices C and D, respectively.

#### 3.2.3 Formative assessment tasks

Over the semester, each learner completed approximately 12 quizzes, 12 homework assignments, and 8 participation evaluations. Each quiz consisted of 20 items (15 multiple-choice and 5 short-answer items). One point was awarded per item and total scores were converted to a 0–100 scale. To reduce the risk that observed learning gains were due to changes in quiz difficulty rather than actual progress, a formal equivalence procedure was implemented. All quizzes, including the early quizzes (Weeks1-4) and the late quizzes (Weeks 9–12), were constructed using a shared blueprint that specified the number of items, the distribution of skill domains (30% vocabulary, 40% grammar, and 30% comprehension), the balance between multiple-choice and short-answer formats, etc. After the student-teachers drafted the items, two applied linguistics professors independently checked each quiz for alignment with the blueprint and appropriateness of difficulty. Items that were judged to be misaligned or clearly too easy or too difficult were replaced before administration.

In addition, homework tasks were designed to extend classroom learning to simple communicative use. Typical assignments included writing short dialogues or paragraphs using target vocabulary and grammar, completing controlled practice exercises, and responding briefly to listening or reading materials. Homework tasks were marked using a simple four-dimension analytic rubric. Accuracy reflected correct use of vocabulary and grammar, complexity assessed the sophistication of sentence structure, completeness captured whether all parts of the task were fulfilled, and fluency referred to the overall flow and readability. Each dimension was rated on a 0–5 scale, and the combined scores were used as part of the formative assessment cycle.

In addition, classroom participation was evaluated eight times during the semester using a brief rating form completed by the student-teachers after selected lessons. The participation rubric covered two aspects, i.e., (a) attendance, (b) preparation, (c) active engagement in class activities (e.g., answering and asking questions), and (d) contributing to pair- and group-work. Each aspect was rated on a 0–5 scale, yielding a total score from 0 to 20, which was rescaled to a 0–100 score for consistency with quizzes and homework.

Taken together, this combination of regular quizzes, homework, and participation evaluations was intended not only to monitor progress but also to provide a stable facilitative structure in the sense of the DMCs framework. Consistent with findings from English courses in Hong Kong higher education, where hybrid and flipped designs supported emotional regulation, perceived control, and self-efficacy through clearly sequenced activities and feedback (Lo, 2023), the present program employed a structured schedule of assessment to sustain engagement. In addition, the communicative homework tasks were regarded as content-rich activities similar to the ones that Lo and Shi (2024) found to foster sustained motivation and perceived gains through authentic and higher-order work with texts.

### 3.3 Procedure

The teaching procedure followed a reciprocal model based on the framework of Directed Motivational Currents (DMCs). The intervention was structured into four phases. Goal Identification: At the beginning of the semester, learners completed a questionnaire that captured their goals and motivations for studying the selected language. These insights were then used to shape and adapt the teaching plans. Motivational Trigger Analysis: After goal setting, learners participated in semi-structured interviews to reflect on what initially inspired their interest in the foreign language. Responses were audio-recorded, transcribed, and analyzed.

Instructional Implementation and Feedback: Teaching plans were segmented into sequential learning modules with clear subgoals. Formative assessments were conducted periodically to monitor learners’ progress, and individualized feedback was provided. Student-teachers were encouraged to adjust task difficulty to maintain an optimal challenge-skill balance. Post-course Reflection: At the end of the semester, learners participated in individual interviews to reflect on their learning experiences, perceived progress, and the effectiveness of the peer-teaching platform. Student-teachers also took part in interviews about their teaching experiences, perceived professional development, and suggestions for future iterations of the program.

For the qualitative interviews, 20 learners were selected using purposive rather than convenience sampling. Sampling was stratified by target language (French, Japanese, Korean, and German), gender, attendance patterns, and primary motivational orientation, in order to ensure diverse representation across these dimensions. The semi-structured interviews typically lasted 25–35 min and were conducted in quiet rooms on campus. Thematic saturation was judged to have been reached after approximately the eighteenth interview, when no substantively new codes or themes emerged; the remaining two interviews were used to confirm this saturation.

### 3.4 Quantitative data analysis

Quantitative data from the questionnaires and assessment tasks were analyzed using SPSS 26.0. Descriptive statistics (frequencies, percentages, means, and standard deviations) were computed for all relevant variables. To examine learning gains, paired-samples t-tests were conducted comparing the average quiz scores from the early part of the semester (first three quizzes) with those from the later part (last three quizzes). In addition, chi-square tests were used to explore associations between categorical variables such as primary motivational orientation and patterns of attendance.

### 3.5 Qualitative data analysis

Interview data were analyzed using a coding framework that was initially developed deductively from the four DMCs dimensions (goal/vision-orientedness, triggering factors and launch, facilitative structure, positive emotionality). As coding progressed, this framework was iteratively refined to incorporate inductive themes emerging from the data (e.g., “peer accountability,” “identity expansion,” “career exploration”).

Two coders first read several transcripts jointly and developed a set of initial coding rules based on the four DMCs dimensions. They then independently coded an initial subset of interviews, compared their coding line by line, and refined code definitions through discussion until consensus was reached. After this training phase, approximately 20% of the transcripts were double-coded independently. Inter-coder reliability for this subset was substantial, with a mean Cohen’s *κ* = 0.73, and theme-level κ values ranging from 0.70 to 0.77 (vision-based goals κ = 0.70; triggering events κ = 0.73; facilitative structure and accountability κ = 0.77; positive emotionality and mutual benefit κ = 0.72), indicating substantial agreement across all themes.

## 4 Results

### 4.1 Quantitative data

**Figure 1:**
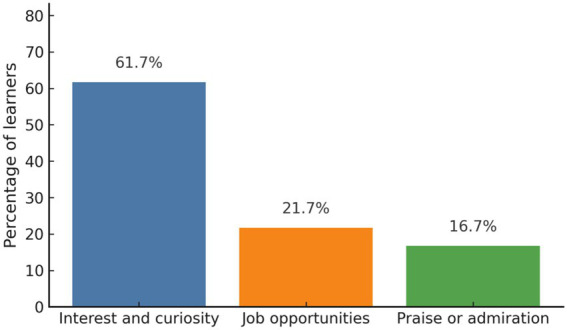
Distribution of participants’ primary learning goals. Learners mainly motivated by curiosity and interest.

Among the 60 student-role participants, 61.7% (*n* = 37) selected interest and curiosity as their primary learning goal, 21.7% (*n* = 13) aimed for future job opportunities, and 16.7% (*n* = 10) were motivated by the desire to be praised or admired (Figure 1). Figure 1 shows that most learners selected interest and curiosity as their primary goal, suggesting a predominantly intrinsic motivational profile.

**Figure 2:**
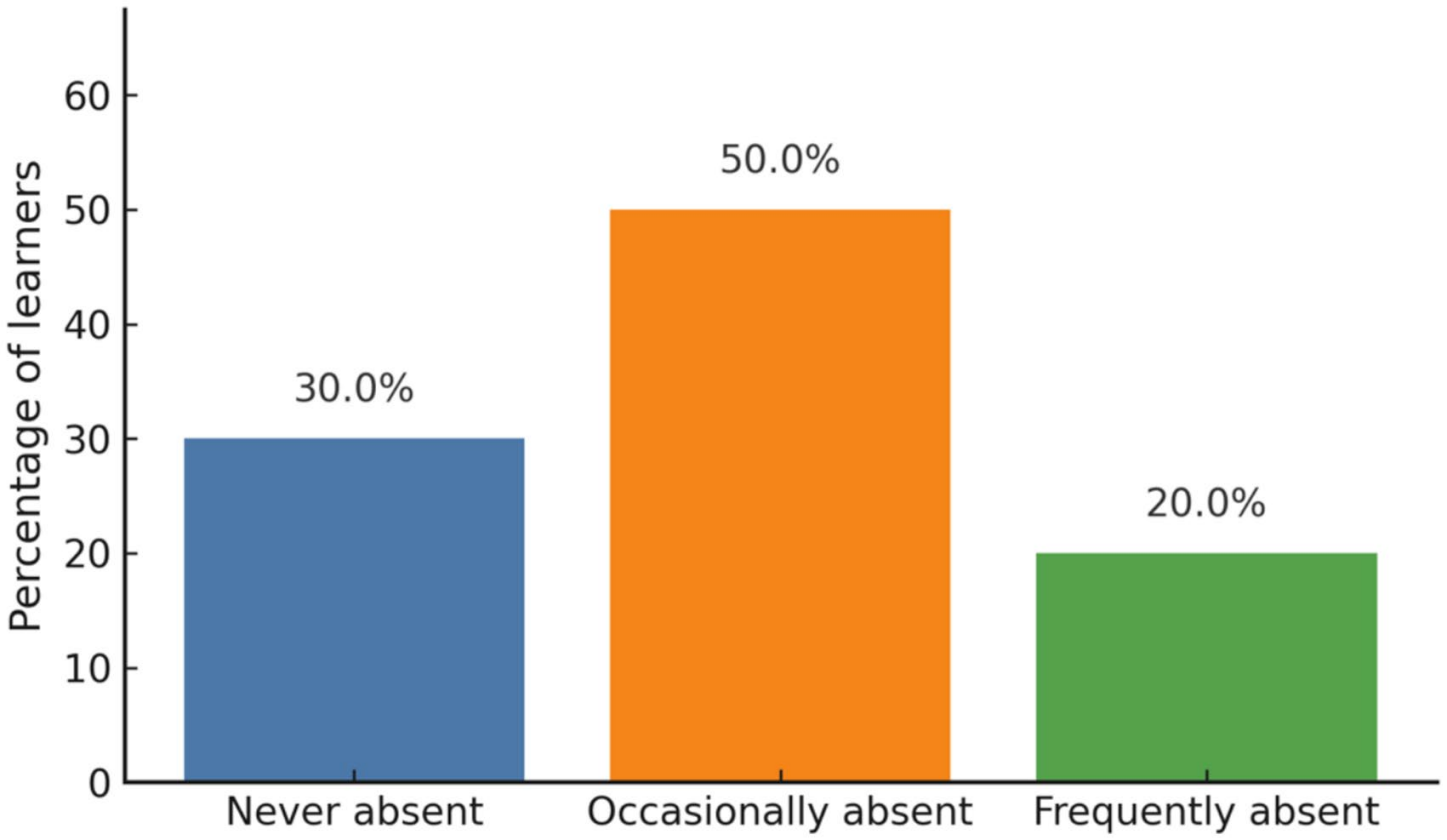
Self-reported attendance frequency. Most learners reported occasional or frequent class absences.

In terms of attendance, 30.0% (*n* = 18) of students reported they were never absent, 50.0% (*n* = 30) reported being occasionally absent, and 20.0% (n = 12) reported frequent absences (Figure 2). Figure 2 indicates that half of the learners reported occasional absences, suggesting that irregular attendance was relatively common in the program.

Exploratory analyses examined the association between primary motivational orientation and attendance frequency using a chi-square test of independence. The analysis revealed a statistically significant association between the two variables, *χ*^2^ (4) = 10.09, *p* = 0.039, Cramér’s V = 0.29, 95% CI [0.17, 0.49]. The full contingency table is provided in Appendix E, and all expected cell counts were at least 2. Learners who reported interest and curiosity as their primary motivation tended to show more regular attendance, whereas those motivated by career goals or social recognition were more likely to report occasional or frequent absences.

**Figure 3:**
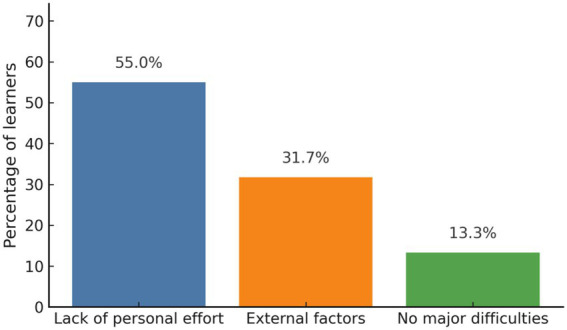
Reported learning difficulties. Most challenges stemmed from limited personal effort.

Regarding learning difficulties, 55% (*n* = 33) attributed their challenges to a lack of personal effort. Of the learners, 31.7% (*n* = 19) cited external factors, such as time constraints or overlapping academic tasks, and 13.3% (*n* = 8) reported facing no major difficulties during the program (Figure 3). Figure 3 reveals that most learners attributed their difficulties to their limited effort rather than external factors, showing an awareness of personal responsibility for sustained learning.

**Figure 4:**
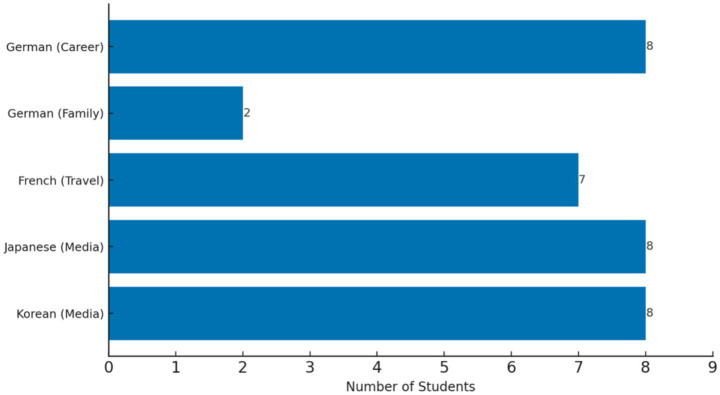
Motivational triggers by language group. Motivational triggers varied across different language groups.

There were clear differences in the distribution of motivational triggers across the four language groups. Among German learners, eight students indicated career goals (e.g., studying abroad or seeking employment in Germany) as their main driver, while two mentioned family influence. French learners primarily cited travel or cultural appreciation (*n* = 7), while Japanese (*n* = 8) and Korean (*n* = 8) learners were mainly motivated by entertainment media such as dramas, and music. These distributions are visualized in Figure 4. Figure 4 indicates that career-related triggers were dominant among German learners, cultural and travel triggers among French learners, and media-related triggers among Japanese and Korean learners, illustrating the diversity of contextual factors that launch DMCs-informed patterns.

**Figure 5:**
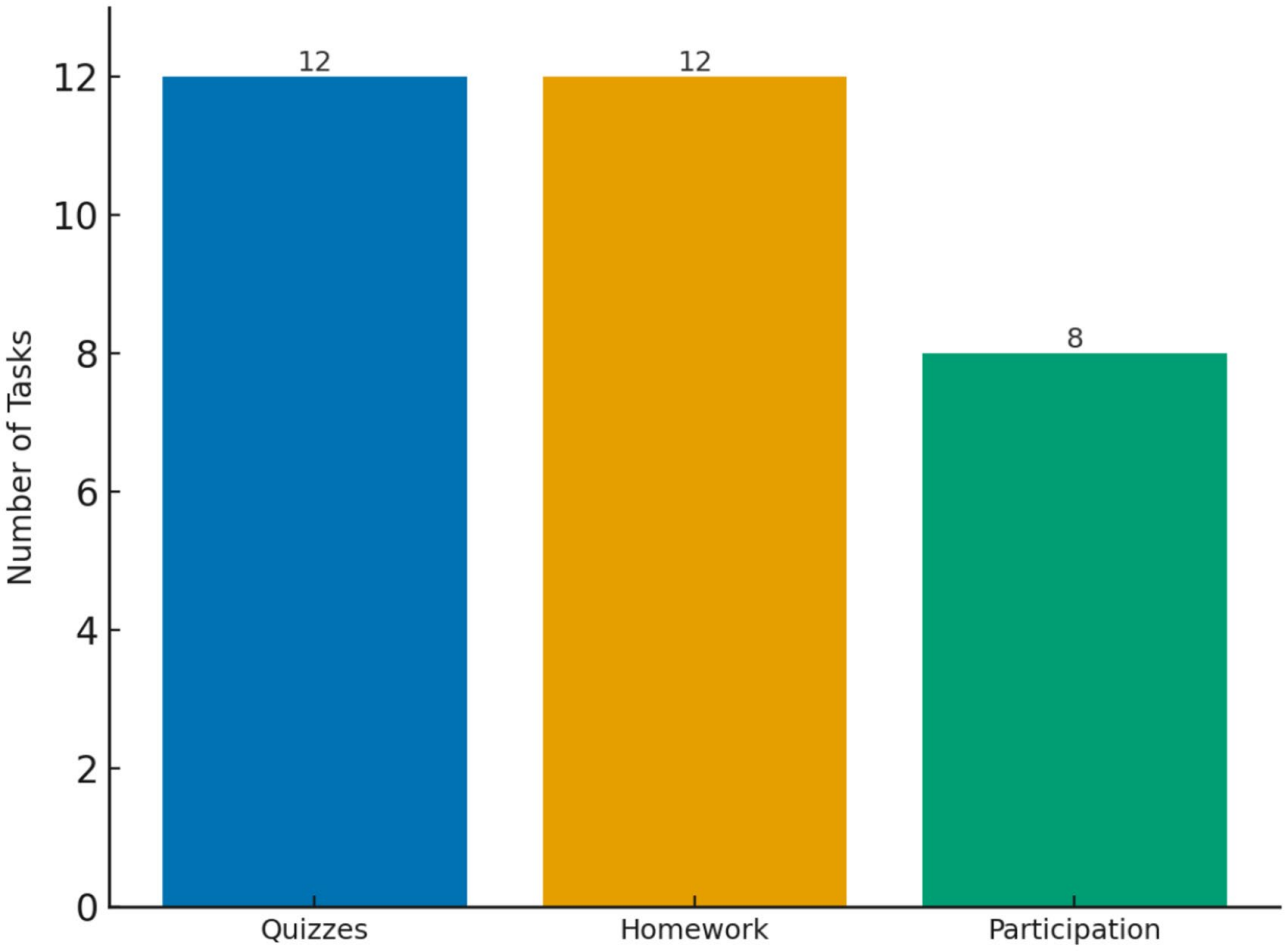
Frequency of assessment components per student. Frequent assessments formed the facilitative structure.

To support learning progress, the program implemented a structured assessment framework. Each student completed approximately 12 weekly in-class quizzes, 12 homework assignments, and 8 classroom participation evaluations throughout the semester. These components provided frequent formative feedback to reinforce learning and track incremental progress (Figure 5). Figure 5 illustrates the regularity of quizzes, homework, and participation assessments that formed the facilitative structure of the program.

**Figure 6:**
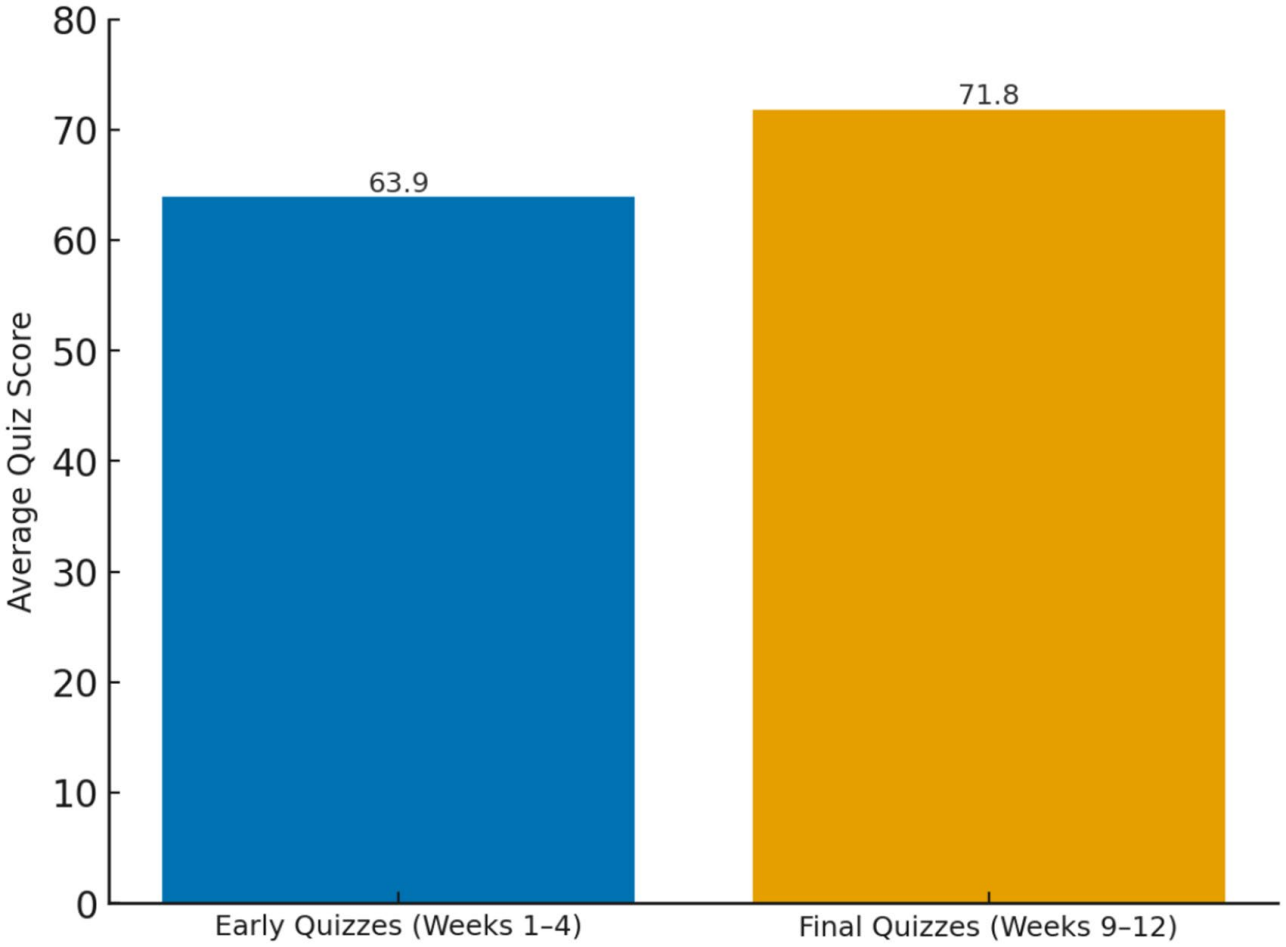
Average quiz scores across early and final weeks. Quiz scores significantly improved over the semester.

A comparison of quiz performance between early and late stages of the semester further indicated measurable improvement. The average score for the first three quizzes (Weeks 1–4) was 63.9 (SD = 6.3), which increased to 71.8 (SD = 5.9) during the final three quizzes (Weeks 9–12). This 7.9-point gain represents a 12.4% relative increase, reflecting a notable improvement in academic performance over time and suggesting that systematic formative assessment contributed to steady learning outcomes (Figure 6). A paired-samples t-test confirmed that the increase in quiz scores was statistically significant, *t* (59) = 10.02, *p* < 0.001, Cohen’s *d* = 1.29, 95% CI [6.30, 9.50]. The Shapiro–Wilk test indicated no violation of normality for the difference scores (*p* = 0.12). Figure 6 shows a clear upward shift in mean quiz scores from the early to the late phase of the semester, indicating consistent improvement in learners’ performance.

**Figure 7:**
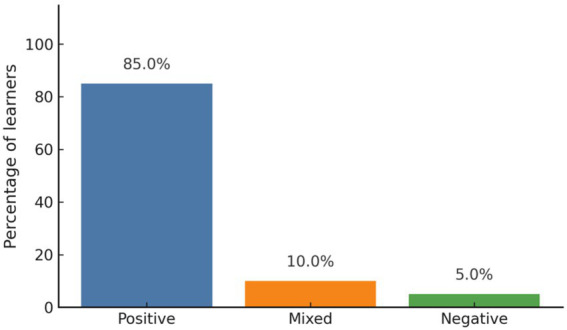
Emotional responses after the program. Positive emotional outcomes predominated after the program.

In terms of emotional outcomes, 85% (*n* = 17) of the 20 interviewed students reported positive emotionality, describing feelings of satisfaction, joy, and self-confidence related to their progress in learning a new foreign language. Ten percent (*n* = 2) reported mixed emotions, and 5% (*n* = 1) expressed negative feelings, mainly due to missing foundational content that hindered later comprehension (Figure 7). Figure 7 indicates that the predominance of positive emotional responses shows that the program successfully fostered the positive emotionality associated with DMCs experiences.

### 4.2 Qualitative themes

The analysis of interview data yielded four major themes aligned with the DMCs framework, i.e., vision-based, intrinsically oriented goals; contextualized triggering events; facilitative structure and mutual accountability; and positive emotionality and mutual benefit. Many learners described vivid visions of themselves as competent language users, as illustrated by comments such as “I imagine myself traveling abroad and talking with locals without needing a translator” (Learner F03, French group) and “I can see myself traveling in Japan and talking freely” (Learner J05, Japanese group). Student-teachers expressed similar future-oriented visions, for example, “I picture myself teaching French professionally 1 day, and this course feels like my first step” (Teacher F01, French group). Such images appeared to stimulate sustained effort.

Learners also identified diverse triggering events. For example, a Korean learner reported, “K-pop brought me here, but news kept me learning” (Learner K02, Korean group), while a German learner mentioned long-term plans to study or work in Germany (Learner G04, German group). Student-teachers likewise referred to concrete triggers, such as “I decided to volunteer because I want to apply for a teaching-related master’s program abroad” (Teacher G02, German group). These narratives resonate with previous accounts of DMCs launches tied to meaningful life events and identity-related goals.

The third theme highlighted the importance of the program’s facilitative structure. Participants noted that weekly quizzes, homework, and participation evaluations helped them develop regular study routines: “Sometimes I felt tired, but because there was a quiz every week, I forced myself to review” (Learner J03, Japanese group) and, as another learner put it, “The weekly quizzes forced me to review even when tired” (Learner K06, Korean group). Student-teachers similarly reported that the need to prepare and evaluate lessons kept them engaged and pushed them to deepen their own language knowledge: “Teaching made me realize how much I’d learned myself” (Teacher F01, French group).

Finally, positive emotionality and mutual benefit were frequently emphasized. Learners described pride and enjoyment when they could successfully use the new language in real or simulated interactions, for example, “When I managed to finish the dialogue without switching to Chinese, I felt really proud of myself” (Learner F01, French group) and “Speaking Korean in role-plays made me feel like I was already abroad” (Learner K05, Korean group). Student-teachers likewise reported increased confidence and a growing sense of professional identity, as reflected in comments such as “Preparing every lesson made me realize I’m capable of being a real teacher in the future” (Teacher J01, Japanese group) and “Teaching confirmed that my own German has become solid enough to help others” (Teacher G02, German group). These themes substantiate the quantitative findings on positive emotional outcomes and show how DMCs-related experiences were shared by both sides of the partnership.

## 5 Discussion

This study investigated a student-led and mutual-benefit foreign language teaching model based on the DMCs framework. Findings revealed that all four DMCs dimensions were actively engaged, contributing to program effectiveness. One of the most salient findings concerns the nature of students’ learning goals. The majority of participants reported that their primary motivation was “interest and curiosity,” indicating a strong orientation toward intrinsic goals. This type of goal is crucial for initiating and sustaining long-term behavior change, as emphasized in the DMCs framework (Dörnyei et al., 2016). Students with such self-driven motivation tended to show more consistent attendance and stronger engagement throughout the course. The significant association between primary motivational orientation and attendance indicates that intrinsically motivated learners were more likely to sustain regular participation over time. In contrast, students with more extrinsic motivations, such as future job opportunities, were more likely to report absences and difficulties maintaining commitment.

This pattern corresponds with Zimmerman’s (1998) model of self-regulated learning, which emphasizes that clearly defined personal goals contribute to proactive learning behaviors across the forethought, performance, and self-reflection phases. Moreover, over half of the students cited their limited personal effort as the main source of learning difficulties. Although some expressed a desire to improve through persistence, others admitted to fluctuating motivation, suggesting that without an internalized goal, learners may struggle to sustain effort over time. In addition to these self-regulatory challenges, roughly one third of learners pointed to external constraints such as time pressure and competing academic workload, while a small minority reported encountering no major difficulties during the program. This supports the work of Rožman and Koren (2013), who argued that successful learning involves a cycle of setting, monitoring, and revising personal goals, especially in tasks requiring extended engagement like language acquisition.

The follow-up interviews further enriched the interpretation of learners’ motivational patterns by revealing highly individualized triggering factors. German learners were primarily driven by career ambitions, including plans for graduate study or employment in Germany. French learners cited cultural fascination and emotional resonance with the language and country, while students learning Japanese and Korean frequently mentioned their interest in popular culture such as K-pop. These findings reflect the diversity of motivational triggers described by Castillo Zaragoza (2011), who emphasized that motivational onset is often rooted in personally meaningful life events. They also align with Başöz and Gümüş’s (2022) categorization of DMCs triggers, including moments of realization, identity-related goals, emotional reactions, and perceived real-world value. Students in this study identified both external circumstances (e.g., media exposure, travel, and family influence) and internal aspirations (e.g., academic vision and self-improvement) as sources of motivation. The presence of such triggering events supports the second DMCs dimension, indicating that a distinct launch point, typically characterized by contextual and cognitive convergence, can initiate sustained engagement (Dörnyei et al., 2016; Henry et al., 2015).

A key factor in sustaining motivation was the facilitative structure of the program, which incorporated progress monitoring, goal segmentation, and behavioral routines. Weekly assessments, structured homework, and participation evaluations helped strengthen students’ sense of progress, creating a semi-automated system of engagement. The 12.4% increase in average quiz scores between the beginning and end of the semester further illustrates this effect. These design choices directly reflect the third DMCs component, facilitative structure, and mirror the function of behavioral routines and subgoal reinforcement described by Dörnyei et al. (2016). Similar patterns have been observed in Hong Kong higher-education English courses in which flipped and hybrid arrangements, combined with clear goal structures and feedback routines, helped students regulate emotions, control their learning, and translate engagement into achievement (Lo, 2023). Students were able to remain motivated in part since they received frequent feedback and observed incremental improvements. The assessment framework was also complemented by motivational strategies to provide real-world communicative exposure. These efforts made language learning not only structured but personally and socially meaningful.

Several classroom episodes are worth highlighting. In one travel-dialogue role-play, French learners prepared conversations for ordering food and asking directions, then performed them in pairs as if they were already abroad; several students reported feeling “as if I was really in Paris” (e.g., Learner F11, F14, F20, French group) and expressed pride when they could complete the exchange without switching to Chinese. In a career-oriented simulation, German learners rehearsed a simple internship interview, which led one participant to remark that this activity made future plans to work in Germany feel “more concrete and achievable” (Learner 07, German group). Across these tasks, learners emphasized that the real-world relevance of the activities made the effort worthwhile and helped them keep attending classes and preparing, even when the linguistic demands were difficult. In this sense, the travel role-plays and career-oriented simulations functioned as content-based and higher-order tasks similar to the literature-focused activities that Lo and Shi (2024) reported to enhance motivation, perceived comprehension, and writing development in a contemporary-fiction course for Hong Kong undergraduates. As noted by Peng and Phakiti (2022), routinized learning environments with real-life relevance are more likely to generate and sustain motivational engagement. Furthermore, the reward and penalty system used in the program (e.g., certificates and small prizes) served as an extrinsic motivational support, which may evolve into autonomous engagement over time, as supported by Steel et al. (2016).

In addition, equally important was the emotional dimension of the learning experience. The majority of interviewed students expressed positive emotionality after completing the program, reporting feelings of joy, satisfaction, and a sense of achievement. These outcomes correspond to the fourth DMCs dimension and align with Ibrahim’s (2016) findings, which suggest that learners experiencing DMCs often report high levels of positive emotionality and a sense of personal growth. These experiences also reflect the concept of eudaimonic well-being (Waterman, 2008), which refers to the psychological fulfillment derived from purpose-driven action. For many students, learning a new foreign language beyond Chinese and English symbolized competence and identity growth. Nevertheless, some participants expressed negative feelings due to missing foundational lessons in pronunciation and grammar. This case illustrates how gaps in early instructional support can result in downward motivational energy, consistent with the case of Özge in Sak (2019), where insufficient instructional supports undermined emotional engagement. A small minority reported negative emotions typically when they had missed foundational lessons or felt that gaps in their knowledge made it difficult to keep up with subsequent content. It further confirms that positive emotionality in DMCs is not guaranteed but must be supported through appropriate pacing and feedback.

Overall, the application of the DMCs framework in constructing a reciprocal and student-led teaching model appeared effective in supporting learners’ engagement and progress. The four dimensions worked together to create a sustainable motivational environment. Students’ learning goals were intrinsically driven and consistent with personal visions; triggers for learning emerged from rich and varied life experiences; facilitative structures were rigorously implemented to guide progress; and positive emotionality reinforced learners’ perceived competence and self-efficacy. The cyclical nature of the model, encompassing investigation, feedback, adjustment, and re-implementation, represents a shift from traditional teacher-centered delivery to a more goal-directed and student-centered paradigm. Furthermore, the program embodied the principle of mutual benefit through its reciprocal teaching-learning design. Student-teachers developed professional competencies and gained recognition, while student-learners got access to meaningful instruction in foreign languages that might otherwise be unavailable to them (Owen and Grealish, 2006; Nabavi et al., 2012).

The present findings can also be situated more explicitly within the broader DMCs literature. Consistent with the systematic review by Jahedizadeh and Al-Hoorie (2021), this study reflects the context-bound nature of many DMCs investigations and indicates how DMCs-informed design can support sustained motivation in a moderate and educationally valuable way rather than inducing excessively intense motivational states. The results also echo Bui’s (2023) study of DMCs in group projects, where carefully structured collaborative tasks and shared goals generated group-level motivational currents among Vietnamese university students. While Bui’s work focused on teacher-designed projects within formal coursework, the current research extends the scope of DMCs applications to a student-led and non-profit platform in which peer teachers and learners jointly create and maintain motivational currents through mutual benefit.

Beyond the DMCs literature, the present findings also resonate with emerging work on English education in Hong Kong higher education. Lo and Chan (2022) showed how the rapid move to online English teaching reshaped teacher-learner mediation and stated that sustainable course designs needed to combine digital tools with clearly structured and student-centered interaction. Lo (2023) further argued that hybrid university English courses could enhance self-efficacy and achievement when they provide emotionally supportive structures, clear expectations, and opportunities for autonomous engagement, while Lo and Shi (2024) demonstrated that authentic and content-based fiction courses could strengthen students’ motivation and perceived gains. Although the present program was implemented in a face-to-face setting, the alignment of the findings of this study with those from Hong Kong studies suggests that peer-led, content-rich, and structurally scaffolded language programs are a feasible way of linking institutional resources, motivational mechanisms, and meaningful tasks in contemporary higher education.

At a theoretical level, the results complement and extend other major motivational frameworks. The prevalence of intrinsic goals and learners’ sense of autonomy align with Self-Determination Theory; the vivid visions of future multilingual self-images resonate with the L2 Motivational Self System; and the dynamic interplay of triggers, structure, and emotional feedback observed in our mixed-methods data corresponds with Complex Dynamic Systems Theory. The DMCs framework unifies these perspectives by emphasizing how such elements converge into a sustained and goal-directed current. The present study shows how DMCs principles could inform the design of an intervention in which a structured pathway directs learners’ initial triggers and visions into consistent study behaviors and positive emotional experiences.

Framed through a simple program evaluation lens, the findings speak to three levels of outcomes, i.e., (a) reaction, reflected in learners’ predominantly positive emotional responses in the post-course questionnaires and interviews; (b) learning, indexed by the significant gains in quiz performance over the semester; and (c) behavior, evidenced by attendance patterns and sustained participation in the weekly sessions. Taken together, these indicators provide preliminary evidence of program impact at multiple levels, while still falling short of a full behavioral or long-term transfer evaluation (e.g., follow-up achievement tests).

To enhance practical relevance, the study also offers several exportable design principles for practitioners who wish to adapt this model. First, structured vision-alignment activities at the beginning can help learners and student-teachers clarify shared and individual goals for the semester. Second, task design should deliberately leverage authentic triggers, such as travel scenarios, media-based activities, or career-oriented simulations, to connect classroom work with learners’ real-world interests. Third, a stable cadence of formative assessment and feedback (e.g., weekly quizzes and homework) is needed to maintain DMC-like currents. Fourth, mechanisms that foster peer accountability and mutual recognition, including clear attendance expectations, shared responsibilities, and acknowledgment of effort and improvement, can strengthen commitment on both sides of the partnership. Finally, it is crucial to put in place basic pacing and support measures, such as offering catch-up opportunities for learners who miss foundational content and keeping class sizes within manageable limits, in order to prevent progressive declines in motivation. Together, these principles provide a concrete basis for adapting DMCs-informed peer teaching in broader educational contexts.

Importantly, the methodological limitations of the sample should be acknowledged. The total number of participants was relatively small and each language group comprised only 15 learners; all eight student-teachers were female and all participants were drawn from a single institution. These features constrain the generalizability of the findings and may limit their applicability to other settings. In addition, since this was a single-arm, pragmatic study without a comparator group, the observed improvements in quiz scores may partially reflect maturation, increasing familiarity with assessment formats, or general instructional quality rather than the DMCs-informed design alone. Thus, the present results are interpreted as preliminary evidence consistent with DMCs principles.

**Figure 8:**
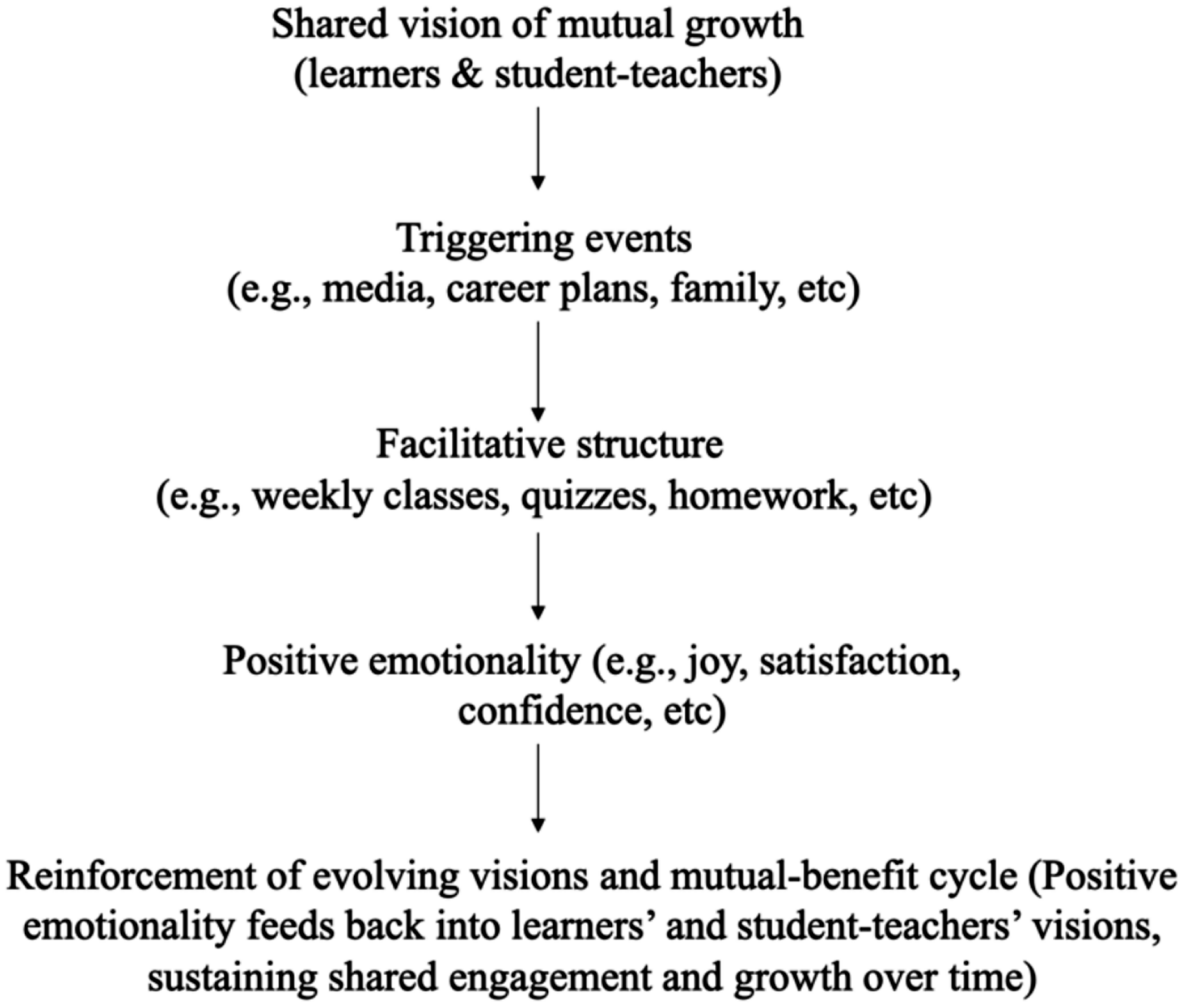
A DMCs-informed mutual-benefit model of the peer-led language program. Interaction of DMCs elements sustaining mutual growth in the peer-led program.

To synthesize these insights, a conceptual model is proposed (Figure 8) that links DMCs elements to reciprocal mechanisms in the present program. At the top of the model is a shared vision of mutual growth, i.e., learners aim to become competent users of a new language, while student-teachers seek to develop teaching skills and a sense of professional identity. Individual motivational currents are launched by diverse triggering events (such as media exposure, career aspirations, or family influences) and are integrated into the program’s facilitative structure (e.g., weekly classes, quizzes, homework, etc.). As learners and student-teachers experience success and recognition, positive emotionality reinforces continued engagement and feeds back into their evolving visions, thereby sustaining the mutual-benefit cycle.

## 6 Conclusion

This study adopted the Directed Motivational Currents (DMCs) framework to design and evaluate a mutual-benefit and non-profit foreign language teaching model in which high-performing language majors volunteered as teachers for undergraduates with limited access to language courses. The model created a reciprocal partnership that supported both pedagogical development for student-teachers and meaningful learning opportunities for student-learners. On the basis of the four DMCs dimensions, i.e., goal/vision-orientedness, triggering factors, facilitative structure, and positive emotionality, the program combined individualized goals with structured routines, regular assessment, and varied motivational strategies. Learners showed measurable gains in quiz performance, indicating that DMCs-informed design can sustain engagement and progress.

Overall, the findings suggest that the DMCs framework is both theoretically applicable and practically useful for constructing learner-centered and sustainable peer-teaching arrangements. The model appears adaptable to other disciplines where peer instruction and systematic scaffolding can promote motivation and achievement. At the same time, the single-arm design, small sample size, and all-female instructor cohort limit the generalizability of the findings, and the learning gains should be interpreted as preliminary rather than as definitive proof of effectiveness. Accordingly, the present results are best viewed as evidence consistent with DMCs-informed peer teaching that should be corroborated by larger, controlled studies in more diverse contexts. Future studies could replicate and extend this work with more diverse settings and longer follow-up periods to examine the durability of DMCs-informed motivation and to compare this approach with interventions grounded in other motivational frameworks.

## Data Availability

The original contributions presented in the study are included in the article/Supplementary material, further inquiries can be directed to the corresponding author.
