# Supplementary material for: Directed motivational currents as a framework for mutual-benefit peer teaching in foreign language education
Source: Front Psychol. 2025 Dec 4;16:1714697. doi: 10.3389/fpsyg.2025.1714697 (PMC12713418; doi:10.3389/fpsyg.2025.1714697)
Supplement: Supplementary file 1 [file Table_1.DOCX]

Appendices

Appendix A. Pre-Course Questionnaire (10 items)

Section 1. Demographics and Background

1. Gender

☐ Male☐ Female☐ Other / Prefer not to say

1. Major and year of study
2. Target language

☐ French☐ Japanese☐ Korean☐ German

Section 2. Learning Motivation and Goals

1. Primary learning goal (single choice)

☐ Interest and curiosity

☐ Future job opportunities

☐ Desire for praise or social recognition

☐ Other (please specify): ___________________

1. Triggering factor for learning this language (single choice)

☐ Media exposure (e.g., K-pop, anime, films)

☐ Career or study plans abroad

☐ Family or friends’ influence

☐ Travel or cultural appreciation

☐ Other (please specify): ___________________

1. I have a clear vision of myself using this language confidently in the future.

(1 = strongly disagree 5 = strongly agree)

1. I feel capable of achieving my language-learning goals with sufficient effort.

(1 = strongly disagree 5 = strongly agree)

Section 3. Anticipated Challenges and Resources

1. I expect to have enough time each week to study this language.

(1 = strongly disagree 5 = strongly agree)

1. I anticipate major difficulties in staying motivated throughout the semester. (reverse-scored)

(1 = strongly disagree 5 = strongly agree)

1. What do you think will be your main difficulty? (open-ended)

Appendix B. Post-Course Questionnaire (10 items)

Section 1. Course Experience and Attendance

1. Attendance frequency (single choice)

☐ Never absent ☐ Occasionally absent ☐ Frequently absent

1. The weekly tasks and quizzes helped me stay on track.

(1 = strongly disagree 5 = strongly agree)

1. I felt motivated and engaged during most of the classes.

(1 = strongly disagree 5 = strongly agree)

1. I often felt excited or proud about my progress.

(1 = strongly disagree 5 = strongly agree)

1. The feedback I received helped me identify specific areas for improvement.

(1 = strongly disagree 5 = strongly agree)

Section 2. Perceived Outcomes and Difficulties

1. Main learning difficulty during the course (single choice)

☐ Lack of effort

☐ External constraints (time/workload)

☐ No major difficulties

☐ Other: ___________________

1. I feel that my ability to use the target language has improved.

(1 = strongly disagree 5 = strongly agree)

1. I plan to continue learning this language after the course.

(1 = strongly disagree 5 = strongly agree)

1. Overall, this course met or exceeded my initial expectations.

(1 = strongly disagree 5 = strongly agree)

1. Emotional response after the program (single choice)

☐ Positive ☐ Mixed ☐ Negative

Appendix C. Learner Interview Protocol

1. What first motivated you to join this program?

2. How has your vision or goal changed during the semester?

3. What specific events or experiences helped you stay motivated?

4. How did weekly quizzes or homework affect your learning routine?

5. What emotions did you feel while participating in the course?

6. Which aspects of the course did you find most helpful or challenging?

7. In what ways did learning with a peer-teacher differ from regular classes?

8. Would you like to continue or recommend this format? Why?

Appendix D. Student-Teacher Interview Protocol

1. Why did you decide to volunteer as a student-teacher?

2. How did preparing lessons influence your own language learning?

3. What strategies helped you maintain learners’ engagement?

4. How did you assess student progress and provide feedback?

5. What emotional experiences stood out for you (positive or negative)?

6. What improvements would you suggest for future iterations?

Appendix E. Contingency Table for χ² Analysis (Motivational Orientation × Attendance Frequency)

| Motivation Type | Never absent | Occasionally absent | Frequent absences | Row total |
| --- | --- | --- | --- | --- |
| Interest / curiosity | 12 | 22 | 3 | 37 |
| Career goals | 4 | 5 | 4 | 13 |
| Social recognition | 2 | 3 | 5 | 10 |
| **Column total** | **18** | **30** | **12** | **60** |

Note. χ² (4) = 10.09, p = .039, Cramér’s V = .29, 95% CI [.17, .49].

All expected cell counts were at least 2.
